# Diarrhæa after Weaning Successfully Treated with Creosote

**Published:** 1846-11

**Authors:** 


"*Diarrhœa after weaning successfully treated with Creosote. By J. A. Mayes, M. D., of Sumpter District, S. Carolina.*—Case I.—Jessie, a little girl aged about 15 months, was attacked in April last with diarrhœa, a short time after weaning. She had previously enjoyed good health, and was as lively and fond of play as children of her age usually are. The diarrhœa was easily controlled by the usual remedies; but she was much weakened and reduced in flesh. An astringent and tonic regimen was prescribed for a few days after the more urgent symptons were relieved. She did not, however, gain in flesh or strength, but gradually became leaner. In the course of a fortnight fever supervened, with great irritability of stomach, vomiting everything as soon as swallowed. The fever was marked by regular exacerbations and intermissions. Quinine enemata in a few days arrested the fever; these being the only means that could possibly be used, the irritability of the stomach being excessive. There was throughout the fever no pain or tenderness on pressure of the stomach or bowels, and the existence of gastro-enteritis was, therefore, thought doubtful. Water or tea acidulated with tartaric acid, after a few days, could be retained; and by using acidulated drinks, elixir vitriol particularly, and the phosphate of iron, the appetite improved somewhat, but the emaciation still increased gradually. The bowels were generally in a very bad state. For a month she continued in this situation, when her appetite failed, and was growing rapidly worse. Being re-called to prescribe for her, I found her symptoms and appearance as follows:—Pale, leucophlegmatic countenance; abdomen tumid and very hot, complaining of much pain under pressure; stools excessively fœtid and dark colored, also frequent; constant harassing dry cough; great emaciation, so much so that the integument on the extremities seemed sufficient for a second covering; no appetite at all, and some irritability of stomach; cold drinks could be retained, but everything else was refused.—This assemblage of symptoms was indicative of a fatal termination of the case, and that speedily, unless some powerful remedy could arrest the progress of the disease. *Prescription*—R. Creosote, 5 drops; loaf sugar, 1 drachm; gum arabic, 1 drachm; water, 2 ounces. Mix intimately. Of this mixture, a tea-spoonful was administered three times daily; at the same time the tepid bath, medicated by an astringent effusion, was used two or three times daily; after a few days the cold bath was used medicated in the same manner. In less than three days the beneficial effects of this treatment were perceptible in the improved appearance of the alvine discharges. Her amendment from this time was rapidly progressive. The last mixture made up for her was—R. Creosote, 6 drops; loaf sugar, gum arabic, aa 1 drachm; carbonate of iron, ½ drachm; water, 4 ounces. Mix. The vial to be well shaken before measuring a dose. A teaspoonful was directed three times a day. After using this mixture, no further medical treatment was thought necessary; but a nourishing diet and exercise advised.

II.—Billy, a little negro child, had the misfortune to be weaned at six months. Being deprived of his natural food, his health soon suffered much. The bowels were very generally out of order, and he soon became very lean. His situation was such that for eight months or more he required constant medical attention. In that time his disease, of course, varied much, being sometimes better, or even nearly well. In the month of January last he became dropsical. The swellings, however, disappeared under a course of chalybeate and vegetable tonics. From his first indisposition, there were great debility and extreme emaciation; impaired appetite, or morbidly-excited appetite; looseness of the bowels; but with little fever.

Having witnessed the excellent effects of creosote in the former case, I determined to give it a trial in this. At the time of prescribing it for him, the emaciation had become even greater than in the other case, but the symptoms generally were not so severe; he had some appetite; his bowels were, as usual, very much disordered. The same prescription was used, and the good result was as plainly visible, though the amendment was not quite so rapid.

At the time of writing this, he takes but little medicine; nourishing diet and exercise seem to be sufficient for his case; and the prospect of a speedy and entire recovery is infinitely better than it has been for ten months."—*Southern Med. and Surg. Journal.*

